# Comparison of compressional and elastic wave simulations for patient-specific planning prior to transcranial photoacoustic-guided neurosurgery

**DOI:** 10.1117/1.JBO.26.7.076006

**Published:** 2021-07-16

**Authors:** Michelle T. Graham, Reese A. Dunne, Muyinatu A. Lediju Bell

**Affiliations:** a Johns Hopkins University, Department of Electrical and Computer Engineering, Baltimore, Maryland, United States; b Mississippi State University, Department of Mechanical Engineering, Mississippi State, Mississippi, United States; c Johns Hopkins University, Department of Biomedical Engineering, Baltimore, Maryland, United States; d Johns Hopkins University, Department of Computer Science, Baltimore, Maryland, United States

**Keywords:** photoacoustic imaging, transcranial imaging, neurosurgery, acoustic simulations, presurgical planning, surgical guidance, photoacoustic-guided surgery

## Abstract

**Significance:** Simulations have the potential to be a powerful tool when planning the placement of photoacoustic imaging system components for surgical guidance. While elastic simulations (which include both compressional and shear waves) are expected to more accurately represent the physical transcranial acoustic wave propagation process, these simulations are more time-consuming and memory-intensive than the compressional-wave-only simulations that our group previously used to identify optimal acoustic windows for transcranial photoacoustic imaging.

**Aim:** We present qualitative and quantitative comparisons of compressional and elastic wave simulations to determine which option is more suitable for preoperative surgical planning.

**Approach:** Compressional and elastic photoacoustic k-Wave simulations were performed based on a computed tomography volume of a human cadaver head. Photoacoustic sources were placed in the locations of the internal carotid arteries and likely positions of neurosurgical instrument tips. Transducers received signals from three previously identified optimal acoustic windows (i.e., the ocular, nasal, and temporal regions). Target detectability, image-based target size estimates, and target-to-instrument distances were measured using the generalized contrast-to-noise ratio (gCNR), resolution, and relative source distances, respectively, for each simulation method.

**Results:** The gCNR was equivalent between compressional and elastic simulations. The areas of the −6  dB contours of point spread functions utilized to measure resolution differed by 0.33 to $3.35\;{\mathrm{mm}}^{2}$. Target-to-instrument distance measurements were within 1.24 mm of the true distances.

**Conclusions:** These results indicate that it is likely sufficient to utilize the less time-consuming, less memory-intensive compressional wave simulations for presurgical planning.

## 1 Introduction

The endonasal transsphenoidal approach to pituitary tumor resection is a minimally invasive technique that requires insertion of surgical instruments through the nose to remove sphenoid bone and underlying pituitary tumors.^1^ Although the procedure is generally safe,^2^ morbidity and mortality rates rise to 14% to 23% and 24% to 26%, respectively, if iatrogenic injury to the internal carotid arteries (ICAs) occurs.^3^^–^^5^ Current intraoperative guidance techniques, such as stereotactic guidance and endoscopy, enable monitoring of the ICAs in close proximity to the surgical site; however, they suffer from two primary limitations. First, stereotactic guidance is subject to registration errors, which can become increasingly large as patient anatomy is disrupted during surgery and deviates from the anatomy in preoperative x-ray computed tomography (CT) or magnetic resonance images. Second, endoscopy is unable to identify the ICAs when they are obscured by bone or other tissues in the operative path. Our group is investigating the use of transcranial photoacoustic imaging as an intraoperative imaging technique for real-time visualization of the ICAs to address these two limitations, as detailed in our original research papers ^6^^–^^11^ and summarized in the literature surveys on this topic.^12^^,^^13^

To achieve photoacoustic imaging for guidance of endonasal transsphenoidal surgery, we propose the insertion of light-transmitting fiber optic devices in the nasal cavity, similar to other surgical tools.^6^^–^^12^ This light source will then excite the hemoglobin within the ICAs, converting the absorbed optical energy to acoustic energy that is received by an externally placed ultrasound receiver. The external ultrasound receiver placement results in a transcranial photoacoustic imaging scenario, which is challenged by acoustic interactions with bone and is known to degrade image quality.^14^^–^^16^ Previous work from our group developed and demonstrated a simulation method to identify naturally occurring acoustic windows in the adult human skull to minimize acoustic interactions with bone and to provide high-contrast photoacoustic images of the ICAs.^10^^,^^11^ We demonstrated that patient-specific simulations have the potential to enable preoperative planning to determine appropriate placement of imaging system components.^10^^,^^11^

**Fig. 1:**
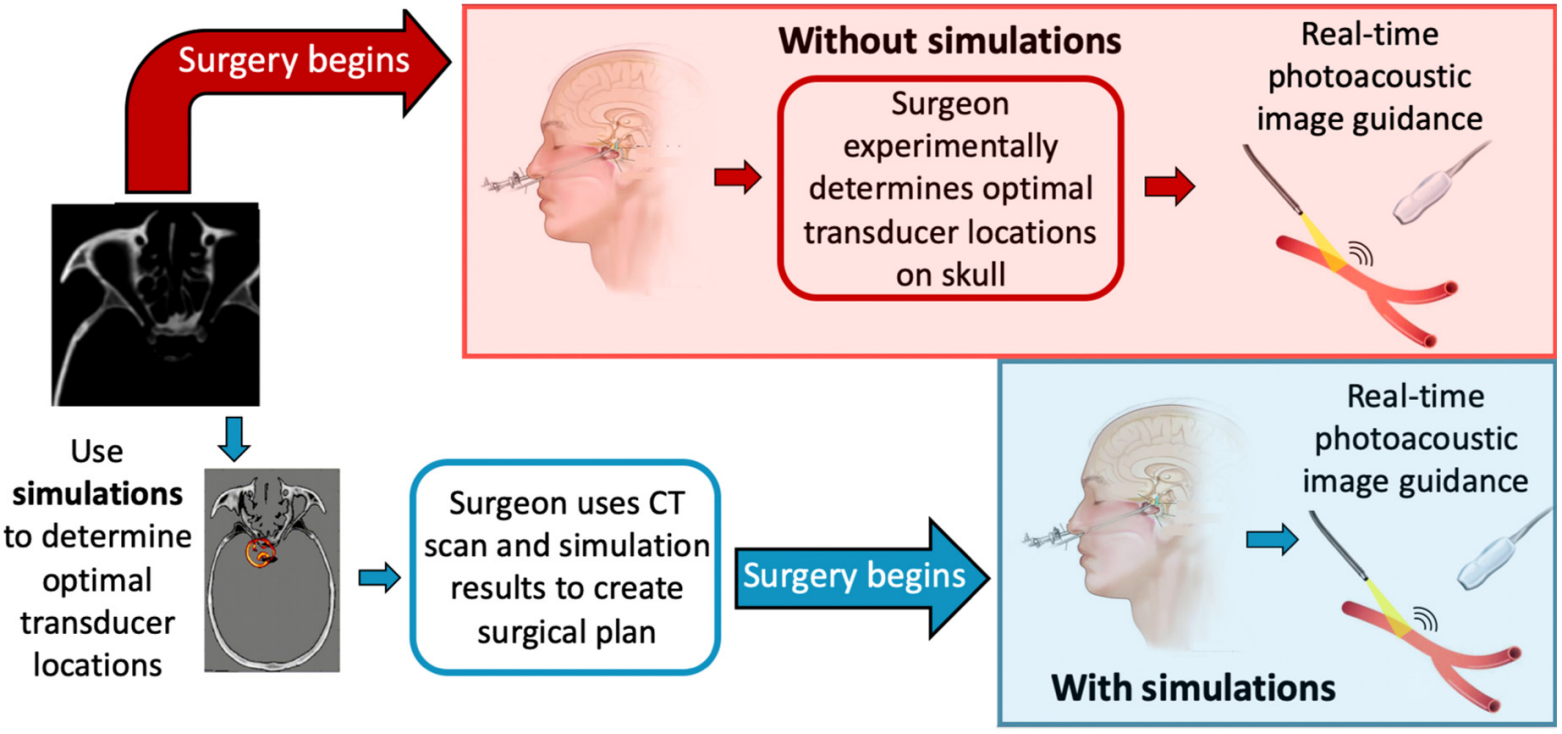
Surgical workflow with and without the use of patient-specific simulations. Without photoacoustic simulations, a surgeon will likely have to test multiple ultrasound receiver locations and their feasibility. Simulations have the potential to efficiently identify these locations before the surgical procedure.

Our vision for a presurgical workflow with the use of simulations is shown in Fig. 1, in direct comparison with a workflow without access to presurgical simulations. Without simulations, a surgeon may need to search and find optimal locations to place an ultrasound receiver to best receive the acoustic signals within the skull. This intraoperative process may be prone to the expenditure of time that could instead be spent operating on the patient and would increase the time that the patient is under anesthesia and on the operating table. In addition, the surgeon may have to abandon the use of intraoperative photoacoustic image guidance during the procedure if a viable receiver location is not determined in time, thereby sacrificing the benefits of real-time photoacoustic-based guidance during the surgery.^10^ In contrast, the simulations that we propose have the potential to preoperatively identify patient-specific ultrasound receiver locations.^10^ The surgeon may then use the simulation results and the patient’s preoperative CT scan to construct a time-efficient surgical plan for each operation. With this step completed, the neurosurgery may then be safely executed with the benefit of real-time, intraoperative photoacoustic image guidance.

Our group previously identified the ocular cavity, temporal region, and nasal cavity as three optimal ultrasound receiver locations, using compressional-wave-only simulations.^10^^,^^11^ However, the shear waves known to propagate within dense media such as bone were excluded from our initial demonstrations. As an alternative, elastic wave simulations, which include both compressional and shear waves, are expected to more accurately represent the physical acoustic process. However, elastic wave simulations, such as the k-Wave elastic simulations based on the classical Kelvin–Voigt absorption model, are time consuming and memory intensive.^17^ Time and memory costs reduce the ease and likelihood of clinical translation.

This paper presents a comparison of compressional and elastic photoacoustic k-Wave simulations to investigate which simulation type (i.e., compressional or elastic) is required for preoperative surgical planning. The remainder of this paper is organized as follows. Section 2 details the simulation methods and quantitative metrics used for the comparison. Section 3 presents the resulting comparisons. Section 4 discusses the implications of the results with respect to the compressional wave simulations that were used in our experimental validation studies, with regard to the vision shown in Fig. 1 and with an eye toward reducing barriers to clinical translation. Finally, Sec. 5 concludes the paper.

## 2 Methods

### 2.1 Simulation Configuration

**Fig. 2:**
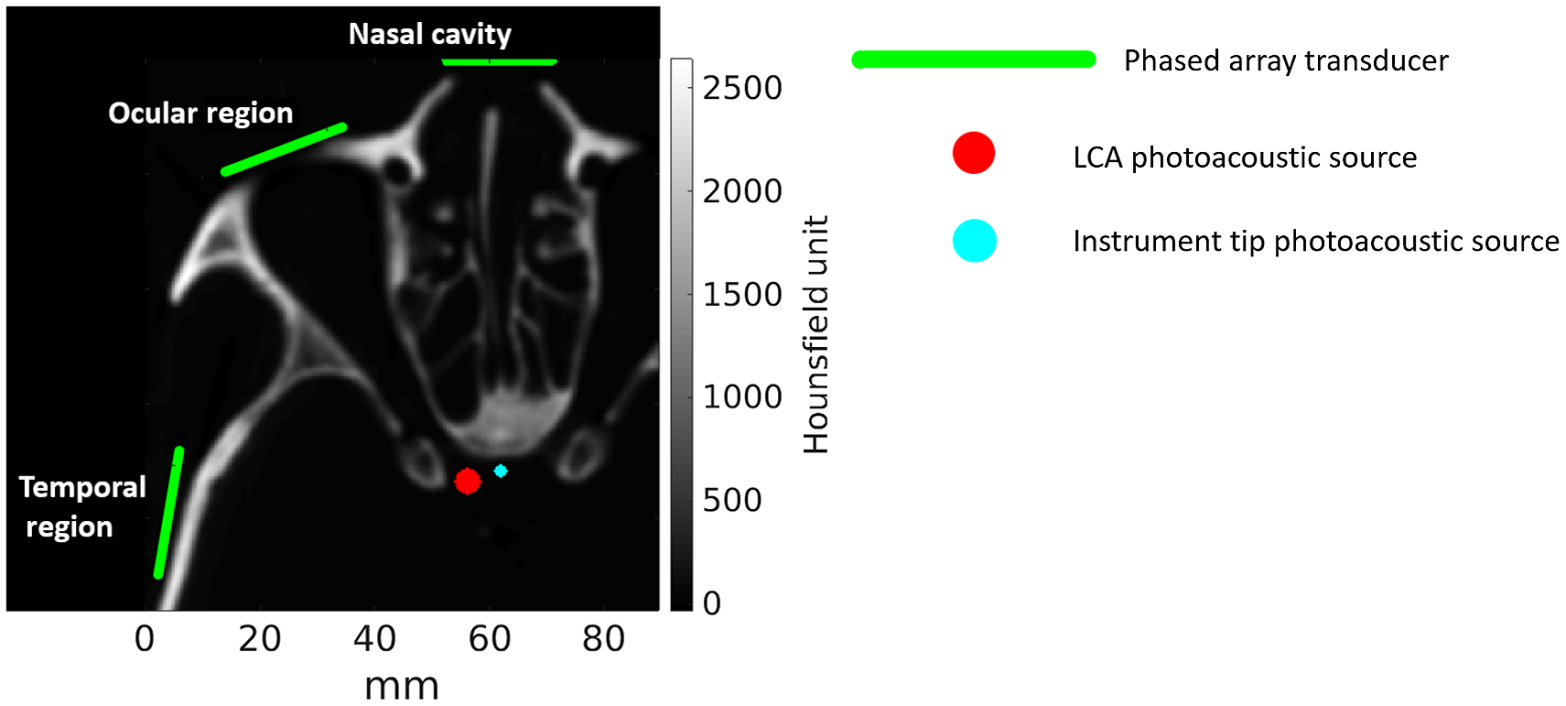
Axial slice from the CT volume of the human cadaver skull demonstrating a 2D cross-section of the 3D simulation configuration. Spherical photoacoustic sources were placed within the LCA and at distances of 6 to 13 mm from the LCA to represent the tip of a surgical instrument (a distance of 6 mm is shown). Green lines illustrate locations of independently placed ultrasound transducers.

**Fig. 3:**
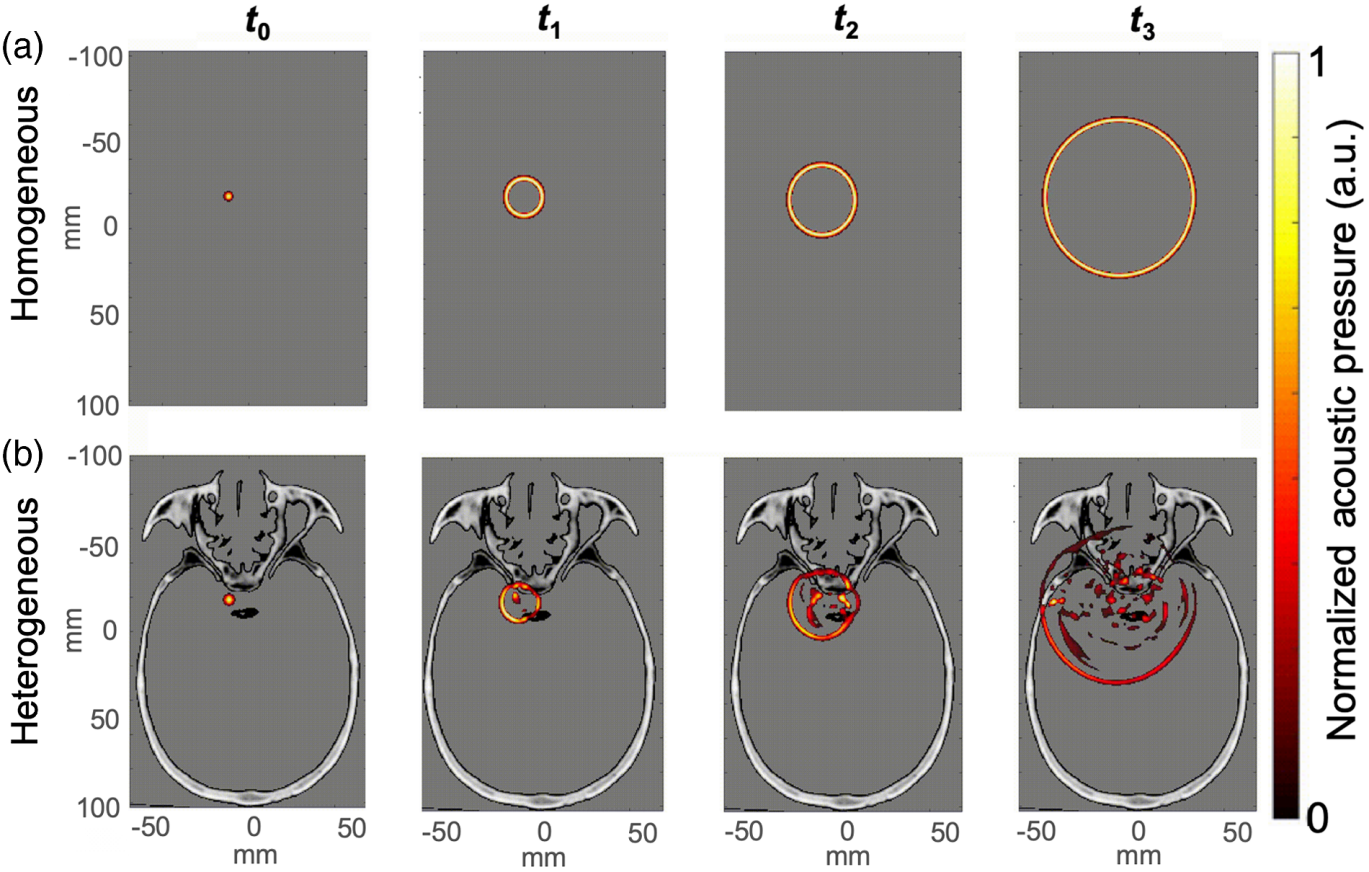
2D cross-section of acoustic wave propagation at various time points (i.e., $t_{0}$ through $t_{3}$) during (a) the homogeneous and (b) heterogeneous 3D simulations. There is no bone present in the homogeneous simulation, as represented by the grayscale background. In both cases, the acoustic wave propagates spherically outward from the initial pressure distribution (location of left ICA). In the heterogeneous simulations, acoustic interactions with cranial bone cause distortions (i.e., aberrations, attenuation, scattering, and reverberations) in the waveform, which ultimately degrade image quality. See Video 1 for the evolution of wave propagation for these two simulations (Video 1: MP4, 0.734 MB [URL: https://doi.org/10.1117/1.JBO.26.7.076006.1]).

Three-dimensional (3D) photoacoustic k-Wave^17^^–^^19^ simulations were performed after converting the CT volume of a human cadaver skull into heterogeneous, volumetric maps of corresponding density, compressional and shear wave sound speeds, and compressional and shear wave absorption prefactors. Figure 2 shows an example axial slice from the CT volume of a human cadaver skull. Homogeneous volumes were additionally modeled as the average density, sound speed, and absorption values of brain tissue to provide baseline photoacoustic images that do not contain heterogeneous tissue effects, such as aberration, attenuation, scattering, and reverberation. The simulated tissue properties for the heterogeneous and homogeneous cases are reported in Table 2. A two-dimensional (2D) cross-section of the propagating wave at various time points for the homogeneous and heterogeneous cases is shown in Fig. 3, with more details available in Video 1. The computational grid was defined with a symmetric voxel size of 0.3  mm×0.3  mm×0.3  mm. Acoustic wave propagation was simulated with a sampling frequency of 82 MHz (i.e., time increment of $1.12^{-8}\;s$) on a NVIDIA Quadro RTX 6000 GPU (Table 1).

**Table 1:** Transcranial photoacoustic simulation parameters.

Simulation parameter	Values
Transducer	
Pitch	0.3 mm
Height	13.6 mm
Kerf	0.0 mm
Elements	64
Bandwidth	84.5%
Center frequency	3.6 MHz
Field of view	90 deg
Simulation grid	
Voxel size	$0.3\times 0.3\times 0.3\;{\mathrm{mm}}^{3}$
Sampling frequency	82 MHz

**Table 2:** Simulated tissue properties of the homogeneous and heterogeneous volumetric maps.^18^^,^^20^^–^^25^ The two shear wave properties of sound speed in brain and absorption power law prefactors in bone and brain are not explicitly known. However, these properties can be estimated as approximately half the corresponding compressional speed of sound and approximately double the corresponding compressional absorption power law prefactor.^24^^,^^26^

		Speed of sound (m/s)	Absorption power law prefactor^19^^,^^27^ ($\mathrm{dB}\;{\mathrm{MHz}}^{1.18}/\mathrm{cm}$)	Density ($\mathrm{kg}/m^{3}$)
Bone	Compression	1300 to 3492^18^^,^^20^	1.36 to 2.50^22^	812 to 2770^18^^,^^21^
	Shear	650 to 1746^24^	2.71 to 5.00^a^	812 to 2770^18^^,^^21^
Brain	Compression	1519^25^	0.21^22^^,^^23^	1045^25^
	Shear	800^a^	0.75^a^	1045^25^

a Estimated shear wave values.

Phased array ultrasound transducers with 0.3 mm pitch, 13.5 mm height, 0 mm kerf, and 64 elements (as reported in Table 1) were positioned to receive transcranial photoacoustic signals from three acoustic windows: (1) the left ocular cavity, (2) the left temple region, and (3) the nasal cavity, as shown in Fig. 2. A spherical photoacoustic source with a diameter of 0.3 mm (i.e., a point source) or 4 mm (i.e., the diameter of a typical adult carotid artery) was placed in the location of the left carotid artery (LCA), as shown in Fig. 2. Background absorption was not modeled in the photoacoustic source distributions for the following two reasons. First, background absorption can be mitigated by carefully selecting the illumination wavelength to preferentially excite the oxygenated hemoglobin over surrounding tissues.^28^ Second, our previous observations in transcranial photoacoustic imaging of the cadaveric ICAs did not display evidence of background absorption.^10^

To investigate photoacoustic image characteristics when the tip of a surgical instrument is also in the imaging plane, a 2-mm-diameter spherical photoacoustic source was positioned within the surgical site at distances of 6 to 13 mm from the 4-mm-diameter LCA target. These distances were measured from the centers of the LCA and instrument targets. Considering this measurement and the best possible system resolution described in Sec. 2.2, the minimum distance simulated was 6 mm. The distances of the LCA source relative to the center of the transducers located on the ocular, nasal, and temple regions were 7.40, 8.02, and 5.03 cm, respectively.

### 2.2 Image Formation and Analysis

Received transducer channel data were bandpass filtered to contain −6  dB frequencies in the range of 1 to 5 MHz. Randomly distributed Gaussian noise was added to the received channel data obtained with the transducer located in the temporal region to model the electronic noise of an imaging system, resulting in a 15-dB channel signal-to-noise ratio (SNR). The same noise distribution was then added to the received channel data obtained with the remaining transducer locations (i.e., nasal and temple regions) to simulate the same noise floor for the three transducer locations, each viewing photoacoustic signals of the same 4 mm target.

Photoacoustic delay-and-sum (DAS) images were generated from the filtered channel data with additive noise. Although advanced beamforming techniques exist to compensate for acoustic heterogeneity,^29^^–^^31^ the DAS beamformer is a more standard choice that was selected to compare acoustic heterogeneity effects between the two simulation types. DAS image quality (i.e., resolution, target visibility, and target detectability) was measured for each transducer location. Resolution was assessed by calculating the area of the −6  dB contour of the point spread function (PSF), measured from images of the point target. The best possible system resolution was also measured as the distance of the minimum cross-section through the center of the −6  dB contour with the smallest area. Target visibility was assessed using images of the the 4-mm-diameter LCA target to measure contrast, SNR, and contrast-to-noise ratio (CNR), while target detectability was assessed using the generalized contrast-to-noise ratio (gCNR).^32^^,^^33^ These image quality metrics are defined as follows:

$$\text{Contrast}=20\;\log_{10}(\frac{\mu_{t}}{\mu_{b}}),$$ (1)

$$\mathrm{SNR}=\frac{\mu_{t}}{\sigma_{b}},$$ (2)

$$\mathrm{CNR}=\frac{\left|\mu_{t}-\mu_{b}\right|}{\sqrt{\sigma_{t}^{2}+\sigma_{b}^{2}}},$$ (3)

$$\mathrm{gCNR}=1-\sum_{k=0}^{N-1}\min\{h_{t}(x_{k}),h_{b}(x_{k})\},$$ (4)

where $\mu_{t}$ and $\mu_{b}$ are the means, $\sigma_{t}$ and $\sigma_{b}$ are the standard deviations, and $h_{t}$ and $h_{b}$ are the histograms of the signal amplitudes within ellipsoidal regions of interest (ROIs) placed within the photoacoustic target (denoted by subscript t) or within the background of the photoacoustic image (denoted by subscript b), N is the number of bins in the histogram, and k is the index of the bin. A total of N=145 bins were used to create the histograms for the gCNR measurements. For each image, a singular target ROI was centered on the brightest pixel in the image. For the images obtained with the ocular and nasal transducer locations, six background ROIs were located around the target ROI to calculate means and standard deviations of image quality metrics. For the images obtained with the temple region transducer location, only five background ROIs were used because the sixth was outside the field of view of the transducer. The areas of the target ROI and each background ROI were equivalent.

Target-to-instrument tip distances were measured from DAS photoacoustic images of the LCA and the instrument tip, calculated as the Euclidean distance between the centroid and each source. The distance error was calculated as the absolute difference between the measured target-to-instrument tip distance and the known target-to-instrument distance.

## 3 Results

Table 3 compares the execution time and memory usage of the 3D compressional and elastic simulations, performed with the three independently placed transducers shown in Fig. 2. The last row of this table reports the mean time and memory usage across the three simulations to demonstrate that elastic simulations required ∼4.6× more time and 2.7× more memory than compressional simulations.

**Table 3:** Comparison of execution times and memory usage for the compressional and elastic k-Wave simulations.

	Time (min)		Memory (GB)	
	Compressional	Elastic	Compressional	Elastic
Ocular	9.26	43.73	3.02	8.10
Nasal	10.24	47.79	3.22	8.65
Temporal	10.65	46.80	3.75	10.13
Mean	10.05	45.85	3.33	8.96

**Fig. 4:**
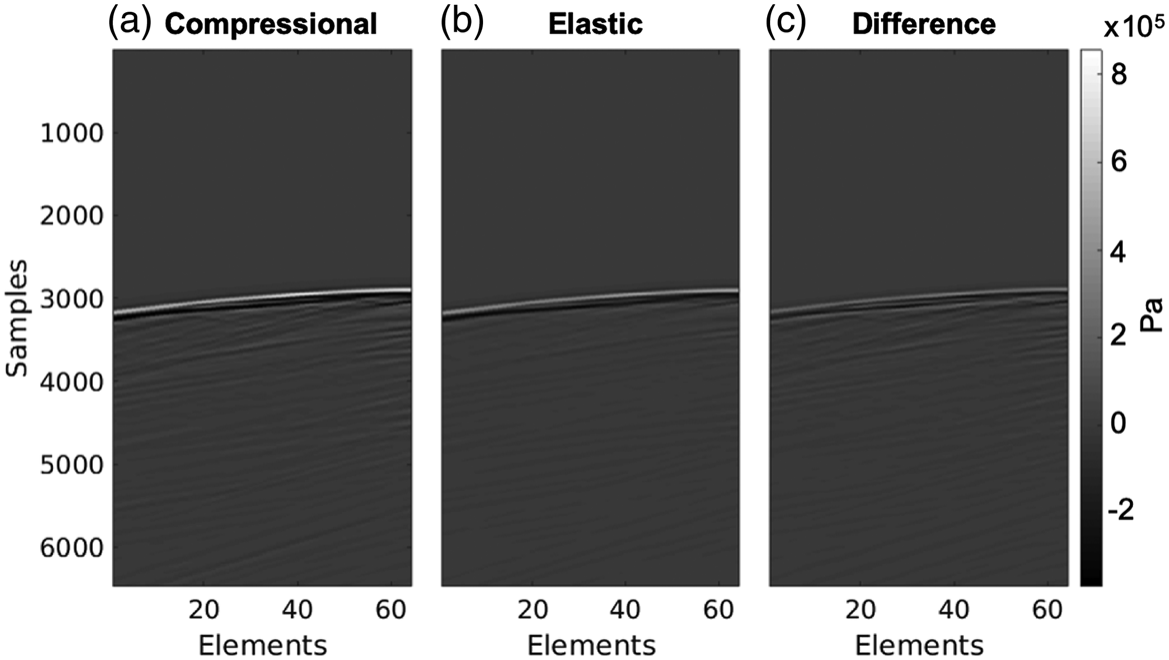
Simulated (a) compressional and (b) elastic photoacoustic channel data of the LCA obtained with the temporal acoustic window. (c) Difference image of the elastic channel data subtracted from the compressional channel data.

Figures 4(a) and 4(b) show simulated photoacoustic channel data from the heterogeneous compressional and elastic simulations of the 0.3-mm-diameter LCA source obtained from the temple acoustic window. Although the initial wavefront shapes across elements look similar between the two simulation types, the compressional simulation has greater reverberations in the photoacoustic signal subsequent to the initial wavefront. To further appreciate the differences in the wavefronts between the simulation types, Fig. 4(c) shows the difference image of the elastic channel data subtracted from the compressional channel data. The wavefront in the compressional simulation has a greater amplitude than the wavefront in the elastic simulation, as indicated by the positive values along the wavefront in the difference image. Specifically, the maximum amplitudes of the wavefronts were $9e^{-5}$ and $6e^{-5}$ Pa for the compressional and elastic simulations, respectively.

**Fig. 5:**
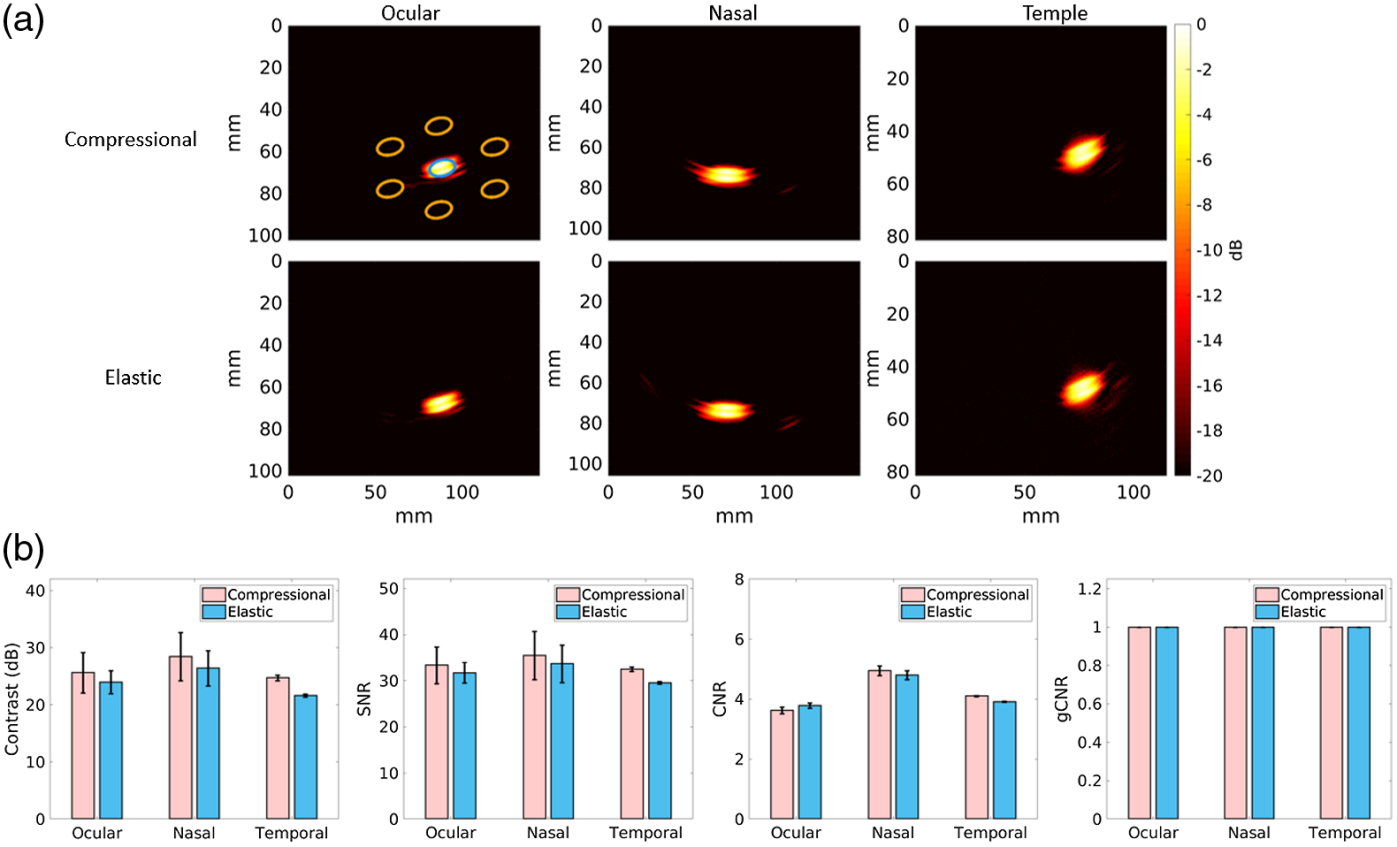
(a) Simulated photoacoustic images of the LCA obtained with the ocular, nasal, and temporal acoustic windows, from left to right, respectively. The top and bottom rows show compressional and elastic simulation results, respectively. The single target and multiple background ellipsoidal ROI are outlined in blue and orange, respectively. Images are displayed with the 10 dB dynamic range. (b) Corresponding mean ± one standard deviation of contrast, SNR, CNR, and gCNR^32^^,^^33^ measurements.

Figure 5(a) shows simulated photoacoustic images generated from the heterogeneous simulations of the 4-mm-diameter LCA source. The shape, location, and visibility of the targets generally agree between the compressional and elastic simulation pairs for each transducer location. Figure 5(b) quantifies the target visibility and detectability of the heterogeneous simulated images displayed in Fig. 5(a). There were minimal differences in contrast, SNR, and CNR measurements, and the gCNR measurements were equivalent. Therefore, the visibility and detectability of these photoacoustic image targets ranges from similar to equivalent for compressional and elastic wave simulations. As noted by Kempski et al.,^33^ there is no benefit to improving particular methods when the gCNR is already near its maximum value of 1.0, and in this case, the method is the type of simulation used for presurgical planning.

**Fig. 6:**
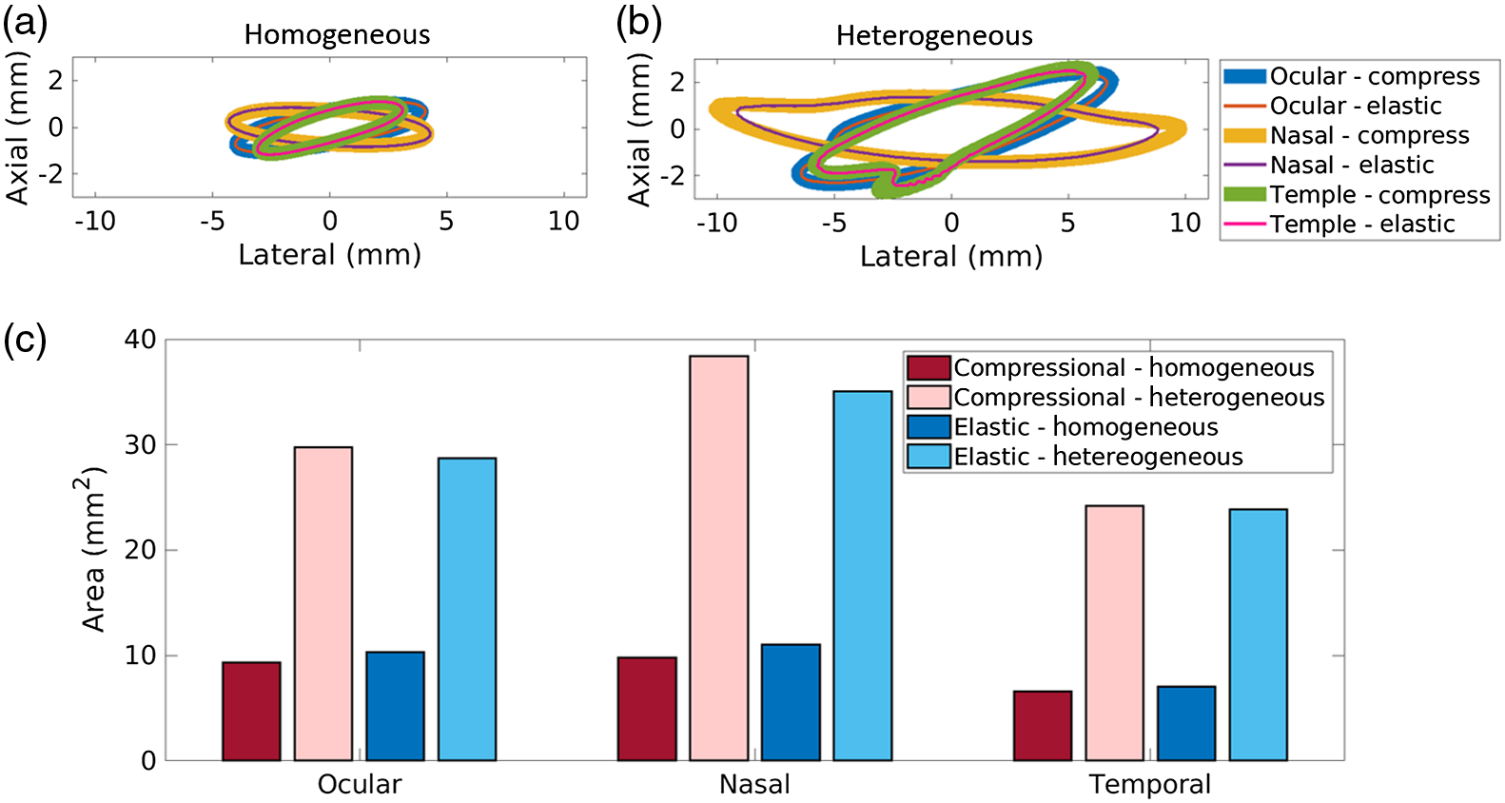
Point spread function −6  dB contours for the (a) homogeneous and (b) heterogeneous point target simulations. (c) Corresponding areas of the PSF −6  dB contours. Homogeneous simulation measurements provide baseline resolution measurements without the negative effects of tissue heterogeneity.

Figures 6(a) and 6(b) show the −6  dB contours of the point target images from the homogeneous and heterogeneous simulations, respectively. The best resolution of the system was 1.4 mm, obtained from the shortest cross-section of the −6  dB contour of the homogeneous compressional simulation image obtained from the temporal region. Figure 6(c) shows the calculated areas of the contours. The contour areas of the baseline homogeneous simulated images were 9.33, 9.80, and $6.60\;{\mathrm{mm}}^{2}$ for the ocular, nasal, and temple acoustic windows, respectively, for compressional simulations and 10.27, 11.05, $7.08\;{\mathrm{mm}}^{2}$, respectively, for elastic simulations. The areas of the corresponding simulated images obtained with the heterogeneous skull model were 29.78, 38.26, and $24.22\;{\mathrm{mm}}^{2}$, respectively, for compressional simulations and 28.76, 35.29, and $24.21\;{\mathrm{mm}}^{2}$, respectively, for elastic simulations. Therefore, the resolution of these photoacoustic images is 0.45 to $1.24\;{\mathrm{mm}}^{2}$ better for the homogeneous compressional wave simulations than the homogeneous elastic wave simulations. However, in the presence of bone, the elastic wave simulations have 0.33 to $3.35\;{\mathrm{mm}}^{2}$ better resolution than compressional wave simulations for the heterogeneous case.

**Fig. 7:**
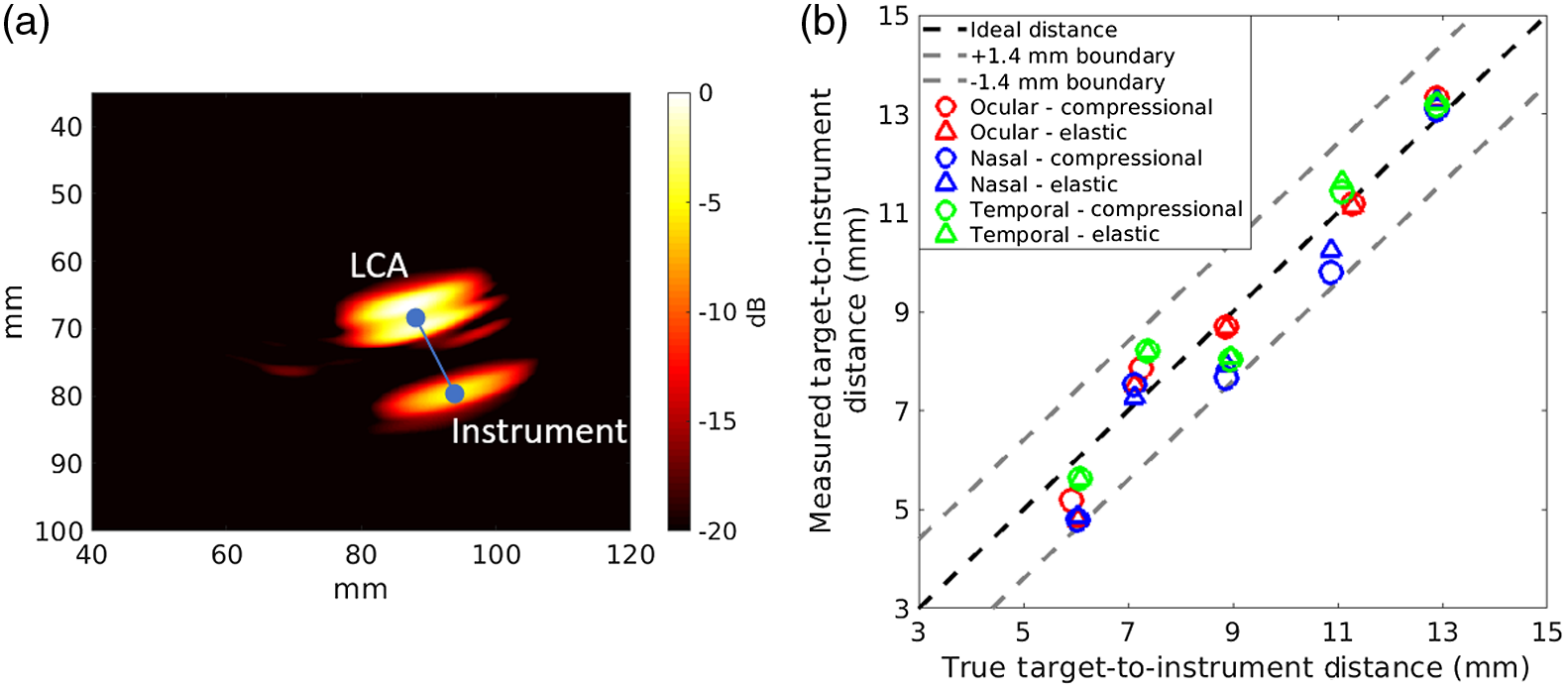
(a) Heterogeneous simulated photoacoustic images of the two sources representing the LCA and instrument tip, obtained with the ocular acoustic window at a relative distance of 11.27 mm. The target-to-instrument distance is the length of the line between the source centers in the image. (b) Comparison of measured target-to-instrument distances as a function of the true target-to-instrument distances for compressional (○) and elastic (Δ) measurements in the ocular, nasal, and temporal acoustic windows. The ideal 1:1 relationship is shown as the dashed black line, with ± 1.4 mm boundaries, representing the best possible resolution of the system, indicated by the dashed gray lines.

Figure 7(a) shows an annotated photoacoustic image from a heterogeneous simulation of the LCA and a surgical instrument tip, obtained with the ocular acoustic window. The ground truth target-to-instrument distance is 11.27 mm, and the measured target-to-instrument distance is 11.18 mm in this image. Figure 7(b) shows image-based measurements of multiple target-to-instrument distances as a function of ground truth distances. The largest distance error between measurements and ground truth was obtained with the nasal cavity images, measuring 1.24 and 1.16 mm for the compressional and elastic wave simulations, respectively.

The error ranges for the ocular and temporal regions were 0.09 to 0.91 mm and 0.14 to 0.88 mm for the compressional and elastic simulations, respectively. When comparing the target-to-instrument distance errors between the compressional and elastic simulations, the differences in errors were 0.03 to 0.09, 0.07 to 0.45, and 0.03 to 0.22 mm for the ocular, nasal, and temporal regions, respectively. Therefore, the relative target-to-instrument distances can be measured with accuracy within the 1.4 mm best possible resolution of the system from both compressional and elastic wave simulations.

## 4 Discussion

This paper details our investigations of k-Wave elastic wave simulations as a method for presurgical planning of transducer placement during transcranial photoacoustic-guided neurosurgery. In particular, we assessed comparative differences in wave propagation, image quality (i.e., target visibility, target detectability, and target size estimation), and target-to-instrument tip distances (i.e., distances between a critical blood vessel and surgical instrument tip) using photoacoustic channel data and images generated from compressional and elastic transcranial simulations, resulting in three key observations. First, wavefronts in the channel data were similar in terms of general wavefront shape, but they differed in amplitude and reverberations between the compressional and elastic simulations, as shown in Fig. 4. Second, target visibility and detectability were qualitatively and quantitatively similar between the compressional and elastic simulations for the three acoustic windows tested, as shown in Fig. 5. Third, the accuracy of target-to-instrument tip distances measured using either compressional or elastic simulations was generally similar between elastic and compressional simulations and was within the best possible resolution of the system, as shown in Fig. 7. Although the latter two observations (based on results presented in Figs. 5 and 7) may seem counterintuitive with respect to shear waves being the primary acoustic component during acoustic propagation in bone, we hypothesize that the similarity of target visibility, target detectability, and target-to-instrument distances between the two simulation types is due to the strategic choice of transducer location ^10^ (i.e., the length of acoustic interaction with bone is minimized along the pathway from the LCA source to transducers) and the relatively minimal thickness of bone with respect to the distance between the source and each transducer.

Our three key observations indicate that it is likely sufficient to utilize the less time-consuming, less memory-intensive k-Wave compressional simulations for presurgical planning, confirming that appropriate complexity was included when comparing simulation to experimental results from a human cadaver.^10^ The arguably minimal 0.33 to $3.35\;{\mathrm{mm}}^{2}$ area differences in PSFs between k-Wave compressional and elastic simulations indicate that future investigations surrounding the effects of PSF differences could be useful prior to completely eliminating elastic simulations from the presurgical planning process. In particular, for the three acoustic windows tested, the areas of the −6  dB contours of the PSFs achieved with the compressional wave simulations was up to $1.24\;{\mathrm{mm}}^{2}$ better than that of the elastic wave simulations for the homogeneous case and up to $3.35\;{\mathrm{mm}}^{2}$ worse than that of the elastic simulations for the heterogeneous case, as shown in Fig. 6. A neurosurgeon user study could possibly be performed to determine if these differences in image resolution would substantially alter a neurosurgeon’s presurgical plan.

Note that our observations are specifically catered to simulation results obtained with the k-Wave Toolbox software package. A comparison with multiple other simulation packages^34^^–^^36^ was beyond the scope of this paper. Using the k-Wave simulation package, we demonstrated for the first time that it is likely sufficient to sacrifice the accuracy of elastic wave simulations by solely relying on compressional wave simulations for presurgical planning. Although this sacrifice affects the photoacoustic waveforms sensed by the simulated receivers (as shown in Fig. 4) and image resolution (as shown in Fig. 6), it may not impact the image quality needed to make surgical decisions based on target detectability and target-to-instrument distance. Thus, we now have a better understanding of this pathway to advance intraoperative transcranial photoacoustic imaging of the ICAs toward surgical use.

Regarding the benefit of shorter simulation times, though minimally invasive neurosurgeries are rarely emergency procedures, surgeons waiting on simulations to complete before operating on a patient might create bottlenecks in presurgical planning. This issue could be a barrier to entry for this technology in hospitals that might not have access to or financial resources for powerful GPUs. Without the GPUs that we used to accelerate our simulations, elastic wave simulations could take several hours to complete and ultimately disrupt the presurgical workflow. When utilizing compressional wave simulations, which require ∼4.6× less time to simulate, surgeons can avoid these presurgical bottlenecks, regardless of their accessibility to GPUs. In addition, reduced computational memory places less strain on computational resource requirements for presurgical simulations and can enable surgeons to perform multiple patient-specific simulations at once. Thus, when implemented with compressional wave simulations, our proposed presurgical planning step has the potential to rapidly identify patient-specific optimal transducer locations for incorporation into the surgical plan, in support of our vision outlined in Fig. 1.

In summary, presurgical identification of optimal photoacoustic imaging system component locations with compressional wave simulations offers three primary benefits. First, this presurgical identification would eliminate wasting valuable operating room time to search for and find a suitable transducer location, thereby reducing the time that the patient is under anesthesia, the total procedure duration, and the medical cost. The second benefit is removal of the potential barrier that the surgeon may be unable to identify a viable transducer location and therefore unable to use photoacoustic image-guidance during the procedure. The third benefit is identification of transducer locations that would minimize image quality degradation from the presence of bone and thereby produce photoacoustic images of the ICAs with the best image quality possible for the patient. Possible future work includes a comparison of simulation types when modeling more complex source distributions, such as the possibility for optical absorption from the deoxygenated blood in the endothelial tubes of the cavernous sinus, and followup neurosurgeon user studies.

## 5 Conclusions

The work presented in this paper is the first to reveal that the less time-consuming and less memory-intensive compressional k-Wave simulations are likely sufficient for identification of optimal transducer locations for transcranial photoacoustic-guided surgery. This assessment is based on target visibility and detectability (e.g., target contrast, SNR, CNR, and gCNR) and relative source-to-instrument distance producing similar or identical quantitative measurements for compressional and elastic simulations. These results have multiple implications for reducing barriers for clinical translation of simulation-based photoacoustic-guided surgery.

## References

[1] Cappabianca P. Cavallo L. M. de Divitiis E., “Endoscopic endonasal transsphenoidal surgery,” Neurosurgery 55, 933–941 (2004). NEQUEB 10.1227/01.NEU.0000137330.02549.0D 15458602

[2] Ciric I. et al., “Complications of transsphenoidal surgery: results of a national survey, review of the literature, and personal experience,” Neurosurgery 40(2), 225–237 (1997). NEQUEB 10.1097/00006123-199702000-00001 9007854

[3] Perry M. O. Snyder W. H. Thal E. R., “Carotid artery injuries caused by blunt trauma,” Ann. Surg. 192(1), 74–77 (1980). 10.1097/00000658-198007000-00013 7406566 PMC1344809

[r4] Raymond J. et al., “Arterial injuries in transsphenoidal surgery for pituitary adenoma; the role of angiography and endovascular treatment,” Am. J. Neuroradiol. 18(4), 655–665 (1997). 9127026 PMC8338477

[5] Valentine R. Wormald P.-J., “Carotid artery injury after endonasal surgery,” Otolaryngol. Clin. North Am. 44(5), 1059–1079 (2011). 10.1016/j.otc.2011.06.009 21978896

[6] Bell M. A. L. et al., “Localization of transcranial targets for photoacoustic-guided endonasal surgeries,” Photoacoustics 3(2), 78–87 (2015). 10.1016/j.pacs.2015.05.002 26236644 PMC4519806

[r7] Bell M. A. L. et al., “Quantifying bone thickness, light transmission, and contrast interrelationships in transcranial photoacoustic imaging,” Proc. SPIE 9323, 93230C (2015). PSISDG 0277-786X 10.1117/12.2078613

[r8] Bell M. A. L. et al., “Experimental assessment of energy requirements and tool tip visibility for photoacoustic-guided endonasal surgery,” Proc. SPIE 9708, 97080D (2016). PSISDG 0277-786X 10.1117/12.2213220

[r9] Bell M. A. L. et al., “Feasibility of transcranial photoacoustic imaging for interventional guidance of endonasal surgeries,” Proc. SPIE 8943, 894307 (2014). PSISDG 0277-786X 10.1117/12.2038511

[r10] Graham M. T. et al., “Simulations and human cadaver head studies to identify optimal acoustic receiver locations for minimally invasive photoacoustic-guided neurosurgery,” Photoacoustics 19, 100183 (2020). 10.1016/j.pacs.2020.100183 32695578 PMC7364163

[r11] Graham M. T. Creighton F. X. Bell M. A. L., “Investigation of acoustic windows for photoacoustic imaging of intracranial blood vessels,” in IEEE Int. Ultrasonics Symp. (IUS), IEEE, pp. 1–4 (2020). 10.1109/IUS46767.2020.9251506

[12] Lediju Bell M. A., “Photoacoustic imaging for surgical guidance: principles, applications, and outlook,” J. Appl. Phys. 128(6), 060904 (2020). JAPIAU 0021-8979 10.1063/5.0018190 32817994 PMC7428347

[13] Wiacek A. Bell M. A. L., “Photoacoustic-guided surgery from head to toe,” Biomed. Opt. Express 12(4), 2079–2117 (2021). BOEICL 2156-7085 10.1364/BOE.417984 33996218 PMC8086464

[14] Kneipp M. et al., “Effects of the murine skull in optoacoustic brain microscopy,” J. Biophotonics 9(1-2), 117–123 (2016). 10.1002/jbio.201400152 25919801

[r15] Pinton G. et al., “Attenuation, scattering, and absorption of ultrasound in the skull bone,” Med. Phys. 39(1), 299–307 (2012). MPHYA6 0094-2405 10.1118/1.3668316 22225300

[16] Estrada H. et al., “Broadband acoustic properties of a murine skull,” Phys. Med. Biol. 61(5), 1932 (2016). PHMBA7 0031-9155 10.1088/0031-9155/61/5/1932 26878583

[17] Firouzi K. et al., “A first-order *k*-space model for elastic wave propagation in heterogeneous media,” J. Acoust. Soc. Am. 132(3), 1271–1283 (2012). JASMAN 0001-4966 10.1121/1.4730897 22978855

[r18] Treeby B. E. Cox B. T., “k-Wave: MATLAB toolbox for the simulation and reconstruction of photoacoustic wave fields,” J. Biomed. Opt. 15(2), 021314 (2010). JBOPFO 1083-3668 10.1117/1.3360308 20459236

[19] Treeby B. E. Cox B. T. Jaros J., “A MATLAB toolbox for the time domain simulation of acoustic wave field, user manual,” Manual Version 1.1, Toolbox Release 1.1 (2016)

[20] Mast T. D., “Empirical relationships between acoustic parameters in human soft tissues,” Acoust. Res. Lett. Online 1(2), 37–42 (2000). ARLOFJ 1529-7853 10.1121/1.1336896

[r21] Schneider U. Pedroni E. Lomax A., “The calibration of CT Hounsfield units for radiotherapy treatment planning,” Phys. Med. Biol. 41(1), 111 (1996). PHMBA7 0031-9155 10.1088/0031-9155/41/1/009 8685250

[r22] Duck F. A., Physical Properties of Tissues: A Comprehensive Reference Book, Academic Press (2013).

[r23] White D. Curry G. Stevenson R., “The acoustic characteristics of the skull,” Ultrasound Med. Biol. 4(3), 225–252 (1978). 10.1016/0301-5629(78)90054-6 751304

[r24] White P. J. Clement G. T. Hynynen K., “Longitudinal and shear mode ultrasound propagation in human skull bone,” Ultrasound Med. Biol. 32(7), 1085–1096 (2006). USMBA3 0301-5629 10.1016/j.ultrasmedbio.2006.03.015 16829322 PMC1560344

[25] Lloyd B. A., Tissue Properties, IT’IS Foundation (2020).

[26] Clement G. T. White P. J. Hynynen K., “Enhanced ultrasound transmission through the human skull using shear mode conversion,” J. Acoust. Soc. Am. 115(3), 1356–1364 (2004). JASMAN 0001-4966 10.1121/1.1645610 15058357

[27] Treeby B. E. Cox B. T., “Modeling power law absorption and dispersion for acoustic propagation using the fractional Laplacian,” J. Acoust. Soc. Am. 127(5), 2741–2748 (2010). JASMAN 0001-4966 10.1121/1.3377056 21117722

[28] Beard P., “Biomedical photoacoustic imaging,” Interface Focus 1(4), 602–631 (2011). 10.1098/rsfs.2011.0028 22866233 PMC3262268

[29] Estrada H. et al., “Virtual craniotomy for high-resolution optoacoustic brain microscopy,” Sci. Rep. 8(1), 1459 (2018). SRCEC3 2045-2322 10.1038/s41598-017-18857-y 29362486 PMC5780415

[r30] Sathyanarayana S. G. et al., “Dictionary learning-based reverberation removal enables depth-resolved photoacoustic microscopy of cortical microvasculature in the mouse brain,” Sci. Rep. 8(1), 985 (2018). SRCEC3 2045-2322 10.1038/s41598-017-18860-3 29343801 PMC5772684

[31] Huang C. et al., “Aberration correction for transcranial photoacoustic tomography of primates employing adjunct image data,” J. Biomed. Opt. 17(6), 066016 (2012). JBOPFO 1083-3668 10.1117/1.JBO.17.6.066016 22734772 PMC3381039

[32] Rodriguez-Molares A. et al., “The generalized contrast-to-noise ratio: a formal definition for lesion detectability,” IEEE Trans. Ultrasonics, Ferroelectr. Freq. Control 67(4), 745–759 (2019). ITUCER 0885-3010 10.1109/TUFFC.2019.2956855 PMC8354776 31796398

[33] Kempski K. M. et al., “Application of the generalized contrast-to-noise ratio to assess photoacoustic image quality,” Biomed. Opt. Express 11(7), 3684–3698 (2020). BOEICL 2156-7085 10.1364/BOE.391026 33014560 PMC7510924

[34] Gao F. Feng X. Zheng Y., “Photoacoustic elastic oscillation and characterization,” Opt. Express 23(16), 20617–20628 (2015). OPEXFF 1094-4087 10.1364/OE.23.020617 26367914

[r35] Poudel J. et al., “Iterative image reconstruction in transcranial photoacoustic tomography based on the elastic wave equation,” Phys. Med. Biol. 65(5), 055009 (2020). PHMBA7 0031-9155 10.1088/1361-6560/ab6b46 31935694 PMC7202377

[36] Kang S. Hwang J., “Tuning the characteristics of photoacoustic pressure in a laser-induced photoacoustic generator: a numerical study,” Appl. Math. Modell. 94, 98–116 (2021). AMMODL 0307-904X 10.1016/j.apm.2020.12.029

